# Extracellular Oxidative Stress Markers in COVID-19 Patients with Diabetes as Co-Morbidity

**DOI:** 10.3390/clinpract12020021

**Published:** 2022-02-28

**Authors:** Devika Sanil Kumar, Gowtham Hanumanram, Prasanna Karthik Suthakaran, Jagadeesan Mohanan, Lal Devayani Vasudevan Nair, Kannan Rajendran

**Affiliations:** 1 Department of Research and Development, Saveetha Medical College and Hospital, Kancheepuram 602 105, Tamilnadu, India; devikasds@gmail.com; 2 Department of General Medicine, Saveetha Medical College and Hospital, Kancheepuram 602 105, Tamilnadu, India; gow.gauti@gmail.com (G.H.); kartpress@gmail.com (P.K.S.); drjagadeesan@gmail.com (J.M.); endork@yahoo.com (K.R.); 3 Department of Paediatrics, Saveetha Medical College and Hospital, Kancheepuram 602 105, Tamilnadu, India

**Keywords:** COVID-19, oxidative stress, superoxide dismutase, glutathione s transferase, acute phase reactants, ferritin, zinc, D-dimer

## Abstract

COVID-19 patients have a higher risk of developing inflammatory responses associated with serious and even fatal respiratory diseases. The role of oxidative stress in exacerbating manifestations in COVID-19 pathogenesis is under-reported.This study aimed touseserum levels of superoxide dismutase (SOD3) and glutathione-S-transferase (GSTp1) by ELISA, zinc (ErbaChem5), ferritin and free iron (VitrosChemistry, Ortho Clinical Diagnosis, Raritan, NJ, USA) at the first encounter of randomly selected RT-PCR-positive COVID-19 patients, for assessing disease severity. The parameters which helped in identifying the severity, leading to poor prognosis, were neutrophil:lymphocyte higher than 4, high CRP, low SOD3 values and high GSTp1 values, and diabetes mellitus as a co-morbidity. Higher zinc levels correlated with high GSTp1 and low SOD3, indicating the protective effect of zinc on ROS. The increased high GSTp1 shows an anticipated protective biochemical response, to mitigate the low SOD3 values due to ROS consumption. Decreased SOD3 levels indicate a state of high oxidative stress at cellular levels, and an anticipated increase in GSTp1 levels points to the pathophysiological bases of increasing severity with age, sex, and co-morbidities, such asdiabetes. High levels of initial GSTp1 and zinc levels possibly offer protection to redox reactions at the cellular level in severe COVID-19 infection, preventing deterioration.

## 1. Introduction

SARS-CoV-2 reminds us of the devastating effects of immune dysregulation, following a possible cytokine storm, blood clotting, and exacerbation of hypoxia—a life-threatening systemic inflammatory syndrome involving elevated levels of circulating cytokines and the hyperactivation of immune cells, which can also be triggered by various pathogens, autoimmune conditions, and some drug therapies. The recognition that such an immune response to the pathogen by our own body, and not the pathogen itself, will result in a multi organ dysfunction has directed our attention to the role of triggering factors which mediate cytokine release. Oxidative stress is increased in severe infections, due to the generation of free radicals which are neutralized by antioxidants through their scavenging power, by stopping chain reactions, peroxide decomposition, metal-chelating, and the induction of antioxidant enzymes [1,2]. In states where free radicals are high, such as in COVID-19 [3], the increased production of reactive oxygen species (ROS) or decreased antioxidants may contribute to the worsening of symptoms, resulting in acute respiratory distress syndrome (ARDS) and multi-organ dysfunction syndrome (MODS) [4].

Oxidative stress is associated with an increase in redox signaling, the stimulation of pro inflammatory factors, and DNA damage [5]. Massive oxidative burst is utilized by the immune cells to kill the invading pathogens. This excessive production of ROS has been demonstrated to have a direct destructive effect on the surrounding cells, resulting in direct lung injury in COVID-19 infection [6]. As a result, pulmonary dysfunction and other MODS that occur after a COVID-19 cytokine storm might be the result of enhanced oxidative stress and innate immune activation working in tandem. The association between the pro-inflammatory cytokine signaling and oxidative stress is still being explored. The destructive effect of ROS not only affects the respiratory epithelium, but also other cells such as RBC, which may also contribute to hypoxia. Free heme and hemoglobin, as a result of hemolysis, can also increase oxidative stress. Excessive ROS generation can also affect the RBC membrane, resulting in its phagocytosis by macrophages and neutrophil [7].

Several viruses cause oxidative stress to aid in their reproduction within the cell [8]. These viruses have evolved the ability to influence—manipulate or suppress—some pathway in their favor, in order to manage ROS levels [9]. In human alveolar type II-like epithelial cells and small airway epithelial cells, respiratory viruses enhance lipid peroxidation and decrease GSH (glutathione), and abrogate the activation of certain pathways, resulting in a reduction in the expression of target genes like super oxide dismutase 1 and 3 (SOD), glutathione peroxidase (GPx), glutathione S-transferase (GST), etc. [10].

SOD constitutes a critical antioxidant defense against oxidative stress in the body, and various studies have demonstrated its therapeutic potential and physiological significance [11,12].

Although we recognize the importance of oxidative stress in the pathophysiology of viral infection and sepsis, its involvement in COVID-19 remains unknown. The measurement of total oxidant capacity for oxidative stress levels remains the main research tool, and is not used routinely clinically. This leads us to think about the role of using GSTp1 (glutathione S-transferase-pi), a cytosolic detoxification enzyme, and SOD3 (extracellular superoxide dismutase-ECSOD) in COVID-19 disease management. Various studies involving COVID-19 have documented the role of SOD1 at cellular levels in the lung; but the lack of easy availability of tissues to investigate SOD1 levels prevents its clinical use. SOD3, though produced from multiple sources and conditions, being predominantly extra cellular, it can be used as an easily available, dependable, accessible biomarker, provided that it can be correlated clinically with disease severity or co-morbidities. Further SOD3 contains Zn, while SOD2 contains manganese. Zn prescription was done in the initial days of management of COVID-19 patients. The role of Zn in diabetes management is also well known. Hence, its effects in the SOD3 levels of COVID-19diabetic patients require further evaluation.

## 2. Materials and Methods

### 2.1. Patient Cohort and Data Collection

This was a cross-sectional study conducted in one of the tertiary care hospitals dedicated to COVID-19 patients in Tamil Nadu, South India, from June 2021–October 2021, when Delta was the predominant strain.

The study population comprised 87 consecutive patients who were diagnosed and admitted due to COVID-19, with or without co-morbidities. Only symptomatic patients were included, and those taking antioxidant supplements were excluded. The patients were subdivided into two groups—<60 years and >60 years, considering the difference in morbidity and mortality in elders, and as mild, moderate, and severe, based on the WHO protocol for disease severity. Their biochemical, hematological parameters were assessed [13]. CT severity rating was also assessed for lung involvement. Collected data included: age, gender, symptoms, co-morbidities, hemoglobin (Hb), neutrophil: lymphocyte ratio (NLR), absolute monocyte count (AMC), erythrocyte sedimentation rate (ESR), C-reactive protein (CRP), ferritin, D-dimer, and lactate dehydrogenase (LDH).

Moreover, 5 mL of blood was collected from the participants, delivered in a lithium heparin container, and the serum was obtained after centrifuging at 15,000 rpm for 5 min. Serum levels of superoxide dismutase (SOD3) and GSTp1 were determined using an enzyme-linked immunoassay (ELISA) purchased from ELK Biotechnology, Wuhan, Hubei, P.R.C, and test-performed according to the specifications by the manufacturer. Zinc (Erba Chem 5), ferritin, and free iron (Vitros Chemistry, Ortho Clinical Diagnosis, Raritan, NJ, USA) were assessed in fully automated analyzers. All methods were done following GCP guidelines, with patients providing written informed consent in accordance with the declaration of Helsinki. Institutional ethical committee approval wasobtained in accordance with institutional requirements, and the study was performed following STROBE guidelines.

### 2.2. Statistical Analysis

SPSS version 21 was used for analysis, and PRISM version-5.03 software for Windows was used for generating graphical representation. Data were analyzed with PRISM version-5.03 software also. The Shapiro–Wilk test was used to determine the data’s normality. A one-way ANOVA was performed with the data of variables that pass the normality test. Data were analyzed for the significance of dependent variables, such as SOD3 and GSTp1 within the disease category—normal but diseased, with co-morbidities, and deceased. Comparisons between groups were performed using a *t*-test and Mann–Whitney non-parametric U test.

## 3. Results

### 3.1. Cohort Characteristics

Eighty-seven patients (37 females and 50 males) with a median age of 49 years (range 15 to 85 years) were enrolled. Fever with upper respiratory infection (77%; N = 67), dyspnoea/tachypnoea (42.5%; N = 37), and anosmia/ hypoxmia (23%; N = 20) were predominant on admission. Moreover, 56% of patients displayed at least two co-morbidities. Upon admission to the COVID-19 ward, patients received supportive therapy, including supplemental oxygen. None of the patients at admission received immune modulatory treatment. All patients underwent chest CT, and the images were examined by an experienced radiologist, as per CT severity scoring (<8/25—Mild; 9–15/25—Moderate; >15/25—Severe).

Since the majority of the patients had varied symptoms, and raised acute phase reactants; with the elderly having more risk possibilities, the patients were sub-divided into two groups—<60 years and ≥60 years—as suggested by National Policy on Older Person, Government of India. The mean N:L ratio was 6.267. Ferritin value was elevated in all patients (normal 17.9—264 mg/dL). Chest CT was done on admission in which 8% (N = 7) were normal, 20.7% (N = 18) were mild, 47.97% (N = 40) were moderate, and 16% (N = 14) were severe. Out of 87 patients, 8 patients died, of which 7 had moderate CT value, and 1 had mild.

### 3.2. Analysis of Age and Gender

The older age group has considerable mortality and morbidity in COVID-19 infection, and hence, a group analysis was conducted based on age, as per National Policy on Older Person, Government of India, according to which, ≥60 years was considered as the elderly population. Moreover, 40.5 years was the median age for the younger group, and 67 years was the median age for the older age group. There were 37 females (<60 year—23 and ≥60 year—14) and 50 males (<60 year—35, ≥60 year—15). The median neutrophil:lymphocyte for <60 year was 4.48 (Range: 0.613—57.23), and ≥60 years was 2.34 (1.082—24.28). There was no significant difference between male and females (NLR—3.64:4.11). The difference in males and females, above and below a 60 years subgroup analysis, shows no significant difference in the above category. The median ferritin level in <60 years was 261 ng/mL and ≥60 year was 201ng/mL, with no significant difference by gender. There was no significant difference in median GSTp1 value with respect to age and gender subgroup analysis. The median GSTp1 value for the <60 years subgroup and total females subgroup was 7.9, and for ≥60 years and total males subgroup, was 7.89 ng/mL. However, there was a significant difference in median values of male and female the ≥60 years subgroup (Male- 7.89; Female- 7.24), and no difference in <60 years subgroup. An age-wise subgroup analysis of SOD3 values showed a significant difference (<60 year—2.875; ≥60 year—0.999; *p* value = 0.0113). There was no significant difference in the male:female subgroup analysis. There was no significant difference between the subgroup analysis by age and gender in both iron and zinc values. However, CRP showed significantly higher median values in the older subgroup (46.1 vs. 24.9; *p*= 0.0063), though there was no difference with respect to sex. An analysis of ESR value showed a wider range (2–120mm/1st hour), with no significant difference between age group and gender. D-dimer values showed a significant difference between both the age and gender subgroups, with 47.37% (36/87) elevated above the upper limit of 246 ng/mL (Table 1).

### 3.3. Analysis of Acute Phase Reactants and Enzymes

The acute phase reactants analyzed were CRP, ferritin, SOD3, GSTp1, and D-dimer. We found abnormal values in all parameters in the enrolled patients. Hence, we divided the 87 patients with available values according to percentiles (Table 2), rather than using the reference value of the local laboratory to ensure the generalizability of the results.

Except the low SOD3 values, all others had high values compared to normal range. Accordingly, the 25th percentile value of GSTp1 was 6.129 ng/mL, CRP—8.7 mg/mL, ferritin—204 ng/mL, iron—35.5 µg/mL. SOD3 value, which was 25th percentile below the normal levels, was 4.2ng/mL. D-dimer values were analyzed by finding the number of patients whose values were above the 25th percentile of upper normal value, as D-dimers were reported to be elevated in all COVID-19 patients, even if asymptomatic. Hence, though the range of D-dimer values for the whole population was 137 to 4200 ng/mL, only those with values more than 285 ng/mL were considered for analysis. Nine (F-5, M-4) were above this 25th percentile value among the elderly population. Moreover, 27 were <60 years of age (F- 14, M-13). Overall, 22/29 (F-11, M-11) of the elderly population had GSTp1 value above the 25th percentile. In the younger age group, 46 (F-21, M-25) had above 25th percentile value, whereas 12 (F-2, M-10) were below it. Iron < 60 years had 38 participants (F-14, M-24) above the 25th percentile and the older group had 24/29 (F-11, M-13). There was no significant difference in male:female ratios of iron levels in both subgroups below the 25th percentile value. An analysis of CRP values showed that ≥60 years (22/29) had above 25th percentile value, with no significant difference in male:female. In ≥60 years, there was no significant difference in number with sex or values. There was asignificant difference in the number of patients above the 25th percentile in the <60 years age group, with 33/58 (F-10, M23) above the 25th percentile value. In the elderly population, no such difference was noted.

### 3.4. Analysis by Co-Morbidities

#### 3.4.1. Analysis of Diabetes with/without Hypertension as Co-morbidity

Diabetes, being the most common non-communicable disease in the subcontinent, and COVID-19, being a state of high inflammatory state with significant oxidative stress, the effects of diabetes on the levels of SOD3 were assessed. The serum levels of parameters were analyzed by one-way ANOVA with SOD3 and GSTp1. There was a significant association (*p* < 0.05) of SOD3 and GSTp1 values with ferritin and Zn in diabetes alone, diabetes with hypertension, and no co-morbidities subgroups (Table 2, Tables S1–S3). When SOD3 values of no co-morbidities, Diabetes and diabetes with hypertension were compared; there was a significance between DM vs no co-morbidities and DM/HT vs. no co-morbidities groups. However, there was no significance in SOD3 values between DM vs. DM/HT group. A similar analysis of GSTp1 values did not yield any significance in all three subgroup comparisons of co-morbidities (Tables S4 and S5).

#### 3.4.2. Subgroup Analysis by Disease Outcome

As far as the disease outcomes were concerned, 70 patients (80.45%) were discharged, while 8 patients (9.2%) were deceased. Of the 56 discharged patients, 5 (7.14%) required admission to ICU before discharge. Among the declared patients, 6 had co-morbidities (diabetes), and 2 had no co-morbidities. An analysis of the deceased group shows a significant correlation between age and D-dimer values (*p*—0.0009), with 5/8 having diabetes as a co-morbidity. Those who were younger than 60 years of age had lower D-dimer value (less than cut off value), irrespective of co-morbidities.

## 4. Discussion

Many studies have reported that co-morbidities, such as cardiovascular disease, diabetes, hypertension, etc., increased mortality and morbidity in COVID-19 patients. Most of them have reported cardiovascular disease as a major co-morbidity [14,15]. However, in a country like India, with a high prevalence rate of diabetes complicating COVID-19, there are not enough studies addressing the pathophysiology/biochemical changes due to this co-morbidity. This study analyzed the effect of diabetes as a co-morbidity on the different age group of COVID-19 patients, and on different parameters, including the association between them, oxidative stress markers, and inflammatory reactants. The median age in this cohort was 59 (20–85) years in the severe group, compared to 48 (15–84) years in the non-severe group (mild and moderate). The present study was conducted when predominant variant spreading in India was of the Delta variant, which is associated with relatively bad prognosis compared to other variants, as described by Pecho-Silva, S., et al. [16]. Among demographic and serological features, several parameters, such as older age and male gender, have been associated with more severe COVID-19 prognosis [15]. Gender affected disease severity on presentation, whereas other patient characteristics, including age, obesity, and immunocompromised state, did not. However, among the patients who died, we could not ascertain any significance with gender, though most of them were diabetic, and glucose levels were under control throughout the treatment. Similar studies have reported an increase in risk for diabetic patients [17]. CT chest done on 87 patients in the study showed no correlation between initial CT values and disease outcome. This is in line with a similar study done on patients with hyperferritinemia, which also did not show any such correlation [18].

The neutrophil lymphocyte ratio was higher than the normal range in the younger age group, and in the elderly, it was within the normal range. Values above 4 were reported to have higher mortality and a predictor of ICU admission in similar studies of COVID-19 patients [19,20].

In this study, serum zinc levels were above normal range, and did not vary with disease severity, or changes in inflammatory markers. This may be due to the fact that homeostatic mechanisms’ serum zinc levels remain stable, even if the dietary intake is low. It decreases in blood only when deficiency is very prolonged [21]. Furthermore, zinc absorption is not regulated in response to changes in zinc status. It depends on a saturable transport mechanism. As the zinc level increases with intake, its fractional absorption declines, and this fractional absorption remains the same as in day 1 of the intake. Even if the facilitated diffusion pathway is saturated, passive diffusion remains, and increases the absorption and blood levels. So, this may be the reason of excess zinc levels seen in the current study of COVID-19 patients. However, decreases in zinc levels were found to be associated with significant mortality [22]. The significant correlation between zinc levels and elevated GSTp1 points to the protective effects of zinc on ROS production at the cellular level, resulting in better prognosis in the present study, even when CT values were grossly abnormal in many, as supported by other studies [23]. Although Mohammed Y et al. had found the levels of zinc to be low in COVID-19 patients, resulting in higher mortality; this finding is not contrary to our study, as many of our patients have been on zinc supplementation already after the first wave of the pandemic [24].

Striking differences in inflammatory parameters (CRP, ferritin) and D-dimer were observed upon admission and during hospitalization between the moderately and severely ill patients, as reported in many studies. Ferritin is not only an inflammatory marker, but also an active player in the “cytokine storm” scenario that characterizes severe COVID-19 [25,26,27]. Ferritin levels in our study do not correlate with prognosis, as is observed by Carubbi et al. [18].

GSTp1 levels were considerably above the normal in all patients. However, a subgroup analysis reveals that males of the younger age group had their GSTp1 levels on the lower side, which correlated with a higher number of deaths in male patients (6/8). This shows that the younger population had a lower protective effect offered by elevated GSTp1, whereas in the older population, co-morbidities played a significant role. Among the patients who died (N = 8), 4 were male, who were <60 years of age, with GSTp1 value lower than the 25th percentile. This shows that the younger population who lacked the protective levels of GSTp1 for overcoming the redox reaction had increased chances of mortality. Kumar P. et al. had found that irrespective of age, especially if older, COVID-19 patients had lower GSH, and increased ROS damage, which may be due to the increased utilization of GSH via the increased expression of GSTp1 in COVID-19, as seen in the present study [28].

Similar to previous studies done by Dworzanski et al. [29] and Muhammad et al. [24], the present study also shows a decrease in SOD3 levels. This is due to the weakened antioxidant system, due to the insufficient antioxidant enzymes, as a result of increased ROS production. Those with co-morbidities such as diabetes and hypertension are in a state of oxidative stress, and infection with COVID-19 increases this oxidative stress, overwhelming the oxidative system, as seen in the present study, with decreased SOD3 and increased GSTp1 levels.

## 5. Conclusions

COVID-19 brings out not only the lacuna in knowledge, but deficiencies in the management of unanticipated viral infections. The use of routine inflammatory markers, with the exception of CRP and D-dimers in severe infections, is limited. Initial ferritin levels do not help in prognostication, and the routine use of trace minerals such as iron, zinc, and multi vitamins is questionable. The available evidence points to the state of high oxidative stress at cellular levels in diabetic COVID-19patients, which can be easily assessed by decreased SOD3 levels—enzymes present extracellularly. An anticipated increase in GSTp1 levels, or replenishing the anti-oxidant, points to the pathophysiological bases of increasing severity with age, sex, and co-morbidities like diabetes. High levels of initial GSTp1 and zinc levels possibly offer protection for redox reactions at the cellular level in severe COVID-19 infection, preventing deterioration.

## Figures and Tables

**Table 1:** Laboratory parameters of COVID-19 patients.

	<60 Years	≥60 Years	*p* Value (*p*< 0.05) *	Females	Males	Females			Males		
	Median (Range) N = 58	Median (Range) N = 29		Median (Range) N = 37	Median (Range) N = 50	Median (Range)			Median (Range)		
						<60 Years N = 23	≥60 Years N = 14	*p* < 0.05 *	< 60 Years N = 35	≥60 Years N = 15	*p* < 0.05 *
**Age (15–85)**	40.5 (15–59)	67 (60–85)	<0.0001 (yes)	37 (15–85)	49 (20–84)	57 (15–59)	65.5 (60–85)	<0.0001 *	41 (20–58)	67 (60–84)	<0.0001 *
**NLR**	4.48 (0.613–57.23)	2.34 (1.08–24.28)	0.1295 (ns)	3.65 (1.18–57.23)	4.11(0.613–32.92)	4.29 (1.18–57.23)	2.22 (1.16–9.58)	0.0583	4.97 (0.613–32.93)	2.46 (1.08–24.28)	0.1806
**CRP (mg/L)**	24.9 (3.5–284.8)	46.1 (1.86–347.6)	0.0063 (yes)	30.7 (1.86–284.8)	31.3 (2.9–347.6)	25.5 (5–284.8)	77.8 (5–235.9)	0.0460 (yes)	23.3 (5–177)	37.1 (2.9–347.6)	0.3094
**ESR (mm/1st hr)**	50 (7–120)	46 (2–120)	0.5531 (ns)	48 (2–120)	48 (12–120)	47 (7–120)	49.5 (2–120)	0.2529	52 (13–120)	39 (20–120)	0.7283
**Ferritin (ng/mL)**	261 (7.9–2470)	201 (40.8–2881)	0.7547 (ns)	223 (16.8–943	223 (7.9–2881)	261 (16.8–840)	201 (61.9–943)	0.0179 (yes)	277 (7.9–2470)	201 (40.8–2881)	0.9478
**GSTp1 (ng/mL)**	7.915 (0.913–10)	7.891 (2.666–10)	0.9964 (ns)	7.9 (0.913–10)	7.891 (1.985–10)	7.915 (0.913–10)	7.891 (2.666–10)	0.9625	7.93 (1.985–10)	7.249 (4.258–10)	0.9915
**SOD3 (ng/mL)**	2.875 (0.064–8.23)	0.999 (0.154–7.451)	0.0113 (yes)	2.54 (0.064–8.23)	2.561 (0.081–8)	2.875 (0.064–8.23)	0.999 (0.324–7.451)	0.2941	2.75 (0.081–8)	1.25 (0.154–5.214)	0.0235 (yes) *
**Iron (µg/dl)**	46 (13–445)	47 (13–117)	0.4684 (ns)	47 (13–445)	47 (13–219)	46 (24–445)	45 (13–117)	0.8879	47 (13–219)	47 (15–104)	0.3093
**Zinc (µg/mL)**	266 (229–324.1)	263 (225 –454)	0.6656 (ns)	265.1 (225–400)	260 (229–454)	266 (241–324.1)	267.9 (251–400)	0.7780	266 (229–312)	273.8 (231–454)	0.3970
**D-dimer (ng/mL)**	279.5 (158–3755)	220 (137–938)	0.2475 (ns)	259 (161–1153)	261 (137–3755)	279.5 (195–1153)	222 (161–938)	0.6926	282 (158–3755)	218 (137–509)	0.4003

Abbreviations: NLR—Neutrophil to lymphocyte ratio, CRP—C-Reactive protein, ESR—Erythrocyte sedimentation rate. N = Number of patients, ns—Not significant; * *p*-value calculated by Mann–Whitney, and a *p* value less than 0.05 was taken.

**Table 2:** Distribution of acute phase reactants and enzymes above 25th percentile.

	Cut Off Value *	≥60 Years				<60 Years			
		Above		Below		Above		Below	
		Female	Male	Female	Male	Female	Male	Female	Male
**GSTp1** **(ng/mL)**	6.129 ng/mL	11	11	3	4	21	25	2	10
**SOD3** **(ng/mL)**	4.2 ng/mL	4	2	12	11	6	10	17	25
**Iron** **(µg/dL)**	35.5 µg/mL	11	13	3	2	14	24	9	11
**Ferritin** **(ng/mL)**	204 ng/mL	7	7	7	8	10	23	13	12
**D-dimer** **(ng/mL)**	285 ng/mL	5	4	9	11	14	13	9	22
**CRP** **(mg/L)**	8.7 mg/mL	12	19	2	4	17	26	5	10

Abbreviations: SOD3—Super oxide dismutase, GSTp1—Glutathione s transferase, CRP—C-Reactive protein; * cut off value—25th percentile values.

## Data Availability

Not applicable.

## Supplementary Materials

The following supporting information can be downloaded at: https://www.mdpi.com/article/10.3390/clinpract12020021/s1, Table S1: Column statistics and One-way ANOVA analysis of Diabetes mellitus as co-morbidity Vs anti oxidant and anti inflammatory parameters; Table S2: Column statistics and One-way ANOVA analysis of Diabetes and Hypertension as co-morbidity Vs anti oxidant and anti inflammatory parameters; Table S3: Column statistics and One-way ANOVA analysis No co-morbidity vs. anti oxidant and anti inflammatory parameters; Table S4: SOD3 levels in co-morbidities DM, NC, DM/HT; Table S5: GSTp1 levels in co-morbidities DM, DM-HT and NC.
