# Supplementary material for: Extracellular Oxidative Stress Markers in COVID-19 Patients with Diabetes as Co-Morbidity
Source: Clin Pract. 2022 Feb 28;12(2):168–76. doi: 10.3390/clinpract12020021 (PMC8938798; doi:10.3390/clinpract12020021)
Supplement: Supplementary file 1 [file clinpract-12-00021-s001.zip › clinpract-1577494-supplementary.pdf]

Supplementary materials

	GSTp1	Iron	Zinc	CRP	Ferritin	SOD3
**Number (n)**	29	29	29	29	29	29
**Minimum**	2.666	13.00	235.3	1.860	16.80	0.0810
**Maximum**	10.00	219.0	400.0	347.6	2881	5.550
**Mean**	7.748	58.52	274.2	101.6	536.6	1.994
**Std. Deviation**	2.077	40.56	33.31	100.4	641.2	1.590
**Std. Error**	0.3858	7.532	6.185	18.65	119.1	0.2953

Bonferroni's Multiple Comparison Test	Mean Diff.	t	P < 0.05	Summary	95% CI of diff
**GSTp1 vs SOD3**	5.754	0.08243	No	ns	-202.1 to 213.6
**GSTp1 vs Iron**	-50.77	0.7273	No	ns	-258.6 to 157.1
**GSTp1 vs ZINC**	-266.4	3.816	Yes	**	-474.3 to -58.54
**GSTp1 vs CRP**	-93.86	1.345	No	ns	-301.7 to 114.0
**GSTp1 vs Ferritin**	-528.9	7.576	Yes	***	-736.7 to -321.0
**SOD3 vs Iron**	-56.52	0.8097	No	ns	-264.4 to 151.3
**SOD3 vs ZINC**	-272.2	3.899	Yes	**	-480.0 to -64.29
**SOD3 vs CRP**	-99.61	1.427	No	ns	-307.5 to 108.3
**SOD3 vs Ferritin**	-534.6	7.659	Yes	***	-742.5 to -326.8

Abbreviations: SOD3- Super oxide dismutase, GSTp1- Glutathione s transferase, CRP- C-Reactive Protein.

Table S1: Column statistics and One-way ANOVA analysis of Diabetes mellitus as co-morbidity Vs anti oxidant and anti inflammatory parameters

	GSTp1	Iron	Zinc	CRP	Ferritin	SOD3
**Number of values**	11	11	11	11	11	11
**Minimum**	4.258	15.00	225.0	5.000	38.50	0.0640
**Maximum**	10.00	445.0	331.0	156.0	940.0	2.630
**Mean**	7.727	89.00	264.8	50.32	310.0	0.9700
**Std. Deviation**	1.692	121.0	27.21	42.62	276.5	0.8256
**Std. Error**	0.5103	36.49	8.205	12.85	83.36	0.2489

Bonferroni's Multiple Comparison Test	Mean Diff.	t	P < 0.05	Summary	95% CI of diff
**SOD3 vs GSTp1**	-6.757	0.1268	No	ns	-169.6 to 156.1
**SOD3 vs Iron**	-88.03	1.652	No	ns	-250.9 to 74.84
**SOD3 vs ZINC**	-263.8	4.953	Yes	***	-426.7 to -101.0
**SOD3 vs CRP**	-49.35	0.9264	No	ns	-212.2 to 113.5
**SOD3 vs Ferritin**	-309.0	5.801	Yes	***	-471.9 to -146.1
**GSTp1 vs Iron**	-81.27	1.526	No	ns	-244.1 to 81.59
**GSTp1 vs ZINC**	-257.1	4.826	Yes	***	-419.9 to -94.22
**GSTp1 vs CRP**	-42.59	0.7995	No	ns	-205.5 to 120.3
**GSTp1 vs Ferritin**	-302.2	5.674	Yes	***	-465.1 to -139.4

Abbreviations: SOD3- Super oxide dismutase, GSTp1- Glutathione s transferase, CRP- C-Reactive Protein.

Table S2: Column statistics and One-way ANOVA analysis of Diabetes and Hypertension as co-morbidity Vs anti oxidant and anti inflammatory parameters

	GSTp1	Iron	Zinc	CRP	Ferritin	SOD3
**No. of values**	39	39	39	39	39	39
**Minimum**	0.9130	13.00	236.4	3.500	7.900	0.5640
**Maximum**	10.00	96.00	338.0	199.0	1300	8.230
**Mean**	7.059	48.15	270.2	33.58	287.0	4.149
**SD**	2.302	21.50	20.81	47.13	314.9	2.401
**Std. Error**	0.3685	3.443	3.333	7.546	50.43	0.3844

Bonferroni's Multiple Comparison Test	Mean Diff.	t	P < 0.05	Summary	95% CI of diff
**GSTp1 vs SOD3**	5.754	0.08243	No	ns	-202.1 to 213.6
**GSTp1 vs Iron**	-50.77	0.7273	No	ns	-258.6 to 157.1
**GSTp1 vs ZINC**	-266.4	3.816	Yes	**	-474.3 to -58.54
**GSTp1 vs CRP**	-93.86	1.345	No	ns	-301.7 to 114.0
**GSTp1 vs Ferritin**	-528.9	7.576	Yes	***	-736.7 to -321.0
**SOD3 vs Iron**	-56.52	0.8097	No	ns	-264.4 to 151.3
**SOD3 vs ZINC**	-272.2	3.899	Yes	**	-480.0 to -64.29
**SOD3 vs CRP**	-99.61	1.427	No	ns	-307.5 to 108.3
**SOD3 vs Ferritin**	-534.6	7.659	Yes	***	-742.5 to -326.8

Abbreviations: SOD3- Super oxide dismutase, GSTp1- Glutathione s transferase, CRP- C-Reactive Protein, SD-Standard deviation

Table S3: Column statistics and One-way ANOVA analysis No co-morbidity vs anti oxidant and anti inflammatory parameters

	DM	NC	DM-HT
**Number of values**	29	39	12
**Minimum**	0.0810	0.5640	0.0640
**Maximum**	5.550	8.230	2.630
**Mean**	1.994	4.149	0.9612
**Std. Deviation**	1.590	2.401	0.7878
**Std. Error**	0.2953	0.3844	0.2274
**Lower 95% CI of mean**	1.389	3.371	0.4606
**Upper 95% CI of mean**	2.599	4.927	1.462

Bonferroni's Multiple Comparison Test	Mean Diff.	t	P < 0.05	Summary	95% CI of diff
DM vs NC	-2.155	4.478	Yes	***	-3.333 to -0.9773
DM vs DM/Ht	1.033	1.533	No	ns	-0.6164 to 2.682
NC vs DM/Ht	3.188	4.920	Yes	***	1.602 to 4.774

Abbreviations: DM-Diabetes, NC-No co-morbidities, HT- hypertension

Table S4: SOD3 levels in co-morbidities DM, NC, DM/HT

Bonferroni's Multiple Comparison Test	Mean Diff.	t	P < 0.05	Summary	95% CI of diff
DM/HT vs NC	0.7698	1.090	No	ns	-0.9580 to 2.498
DM/HT vs DM	0.08069	0.1099	No	ns	-1.716 to 1.877
NC vs DM	-0.6891	1.314	No	ns	-1.972 to 0.5943

Abbreviations: DM-Diabetes, NC-No co-morbidities, HT- hypertension

Table S5: GSTp1 levels in co-morbidities DM, DM-HT and NC
